# Correlation of electroretinography components with visual function and prognosis of central retinal artery occlusion

**DOI:** 10.1038/s41598-020-68957-5

**Published:** 2020-07-22

**Authors:** Hyeong Min Kim, Kyu Hyung Park, Se Joon Woo

**Affiliations:** Department of Ophthalmology, Seoul National University College of Medicine, Seoul National University Bundang Hospital, 173-82 Gumi-ro, Bundang-gu, Seongnam-si, Gyeonggi-do 13620 South Korea

**Keywords:** Retinal diseases, Vision disorders

## Abstract

We investigated the full-field electroretinographic (ERG) parameters with visual function and prognosis in central retinal artery occlusion (CRAO), according to its severity. 110 affected eyes of CRAO patients were enrolled and compared with fellow uninvolved eyes (N = 110) and normal control eyes (N = 30). B/A ratio and photopic negative response amplitude (PhNR) resulted in statistically significant differences among the CRAO subgroups according to the severity of retinal ischemia. Amplitudes of PhNR indicating ganglion cell function showed a more marked decline in mild to severe ischemia than those of the B-wave. In terms of visual function and outcome, baseline visual acuity and visual field defects were correlated with B/A ratio only (both, *P* < .001), whereas improvements in visual acuity and visual field were correlated with B-wave amplitude in dark-adapted 3.0 (*P* = .004 and .006), B/A ratio (*P* = .023 and .008), and PhNR amplitude (*P* < .001 and .004). These three ERG parameters were found to be credible predictive factors of visual prognosis. In conclusion, B-wave amplitude in dark-adapted 3.0, B/A ratio, and PhNR amplitude changes in eyes with CRAO are associated with baseline features related to the severity of retinal ischemia, and these are correlated with visual function and prognosis.

## Introduction

Central retinal artery occlusion (CRAO) is a devastating sudden painless visual loss caused by infarction of the retinal inner layer^1–3^. Most CRAO patients suffer from marked reduction of visual function such as visual acuity (VA) of counting fingers or less, and visual field defects such as central scotoma or temporal island with classic signs of infarction observed in the fundus^4,5^. These clinical visual functions and prognoses are known to be related to the severity of CRAO; in other words, the degree of retinal ischemia categorized as incomplete, subtotal, or total CRAO by Schmidt and colleagues^6^: Incomplete CRAO is characterized by reduced VA, minimal retinal edema with indistinct cherry-red spots found in the fundus photo and OCT, and mildly delayed retinal arterial perfusion on fluorescein angiography (FA); Subtotal CRAO by severe decrease in VA, definite retinal edema with cherry-red spots, and severely delayed retinal arterial perfusion; and Total CRAO by deteriorated VA as to hand motion, light perception or less, massive diffuse retinal edema with disruption of retinal structures accompanied by possible choroidal perfusion delay. In addition, our prior studies have uncovered that the severity of CRAO is strongly related to diminished visual acuity, visual field defect types and impairments, arterial blood flow delay, and lastly treatment results^7–9^.

Electroretinography (ERG), which detects electrical responses from the retinal layers^10,11^, is widely accepted as the relevant examination for measuring the retinal layer function, since the ERG components such as amplitude and implicit time could be analyzed quantitatively^12–14^. Generally, an electronegative ERG is considered the characteristic feature of CRAO. It is described as having the B-wave with a smaller amplitude while the A-wave is preserved or minimally attenuated^14^. However, if the retinal perfusion is diminished but still remains as in incomplete CRAO, a typical electronegative ERG pattern is not likely to be distinct. Moreover, attenuated oscillatory potentials and a delay in light-adapted 3.0 flicker response are observed in CRAO patients^14^. Numerous previous studies have investigated the association between ERG component changes and CRAO^10,11,15–17^. Karpe and Henkes first described electroretinography in the arterial and venous diseases of the retina^10,11^. Machida et al.^16^ and Matsumoto et al.^17^ suggested that photopic negative response (PhNR) was emphasized in retinal circulatory disturbances and reflects severity of CRAO. Yotsukura and Adachi-Usami^15^ demonstrated that B/A ratio was correlated with visual acuity improvement.

Notably, PhNR has become a significant component of the ERG response nowadays. First discovered by Viswanathan et al.^18^, it is acknowledged as the negative-going wave followed by the B-wave of cone response after a brief stimuli. This response is known to reflect the retinal ganglion cell (RGC) function, and hence, PhNR has been widely used for clinical purposes such as evaluation of inner retinal diseases^19^.

In our study, we focused on the changes of ERG parameters in eyes with CRAO according to the degree of retinal ischemia, and investigated their correlation with clinical visual functions such as visual acuity and visual field, prognoses, and their improvement.

## Results

### Clinical characteristics of patients

**Figure 1:**
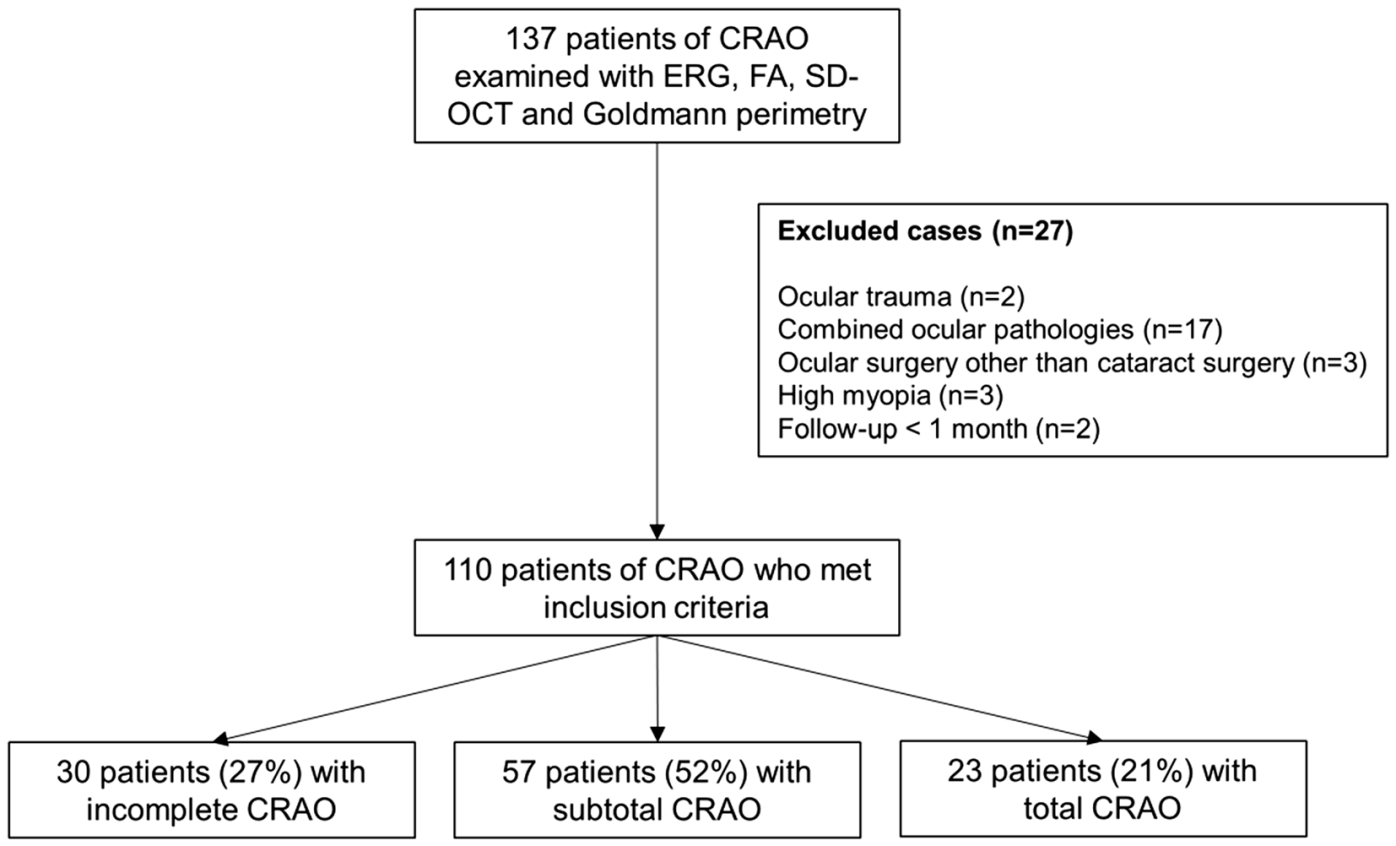
Flow diagram showing the selection process of the enrolled eyes with central retinal artery occlusion. *CRAO* central retinal artery occlusion, *ERG* electroretinogram, *FA* fluorescein angiography, *SD-OCT* spectral domain optical coherence tomography.

**Figure 2:**
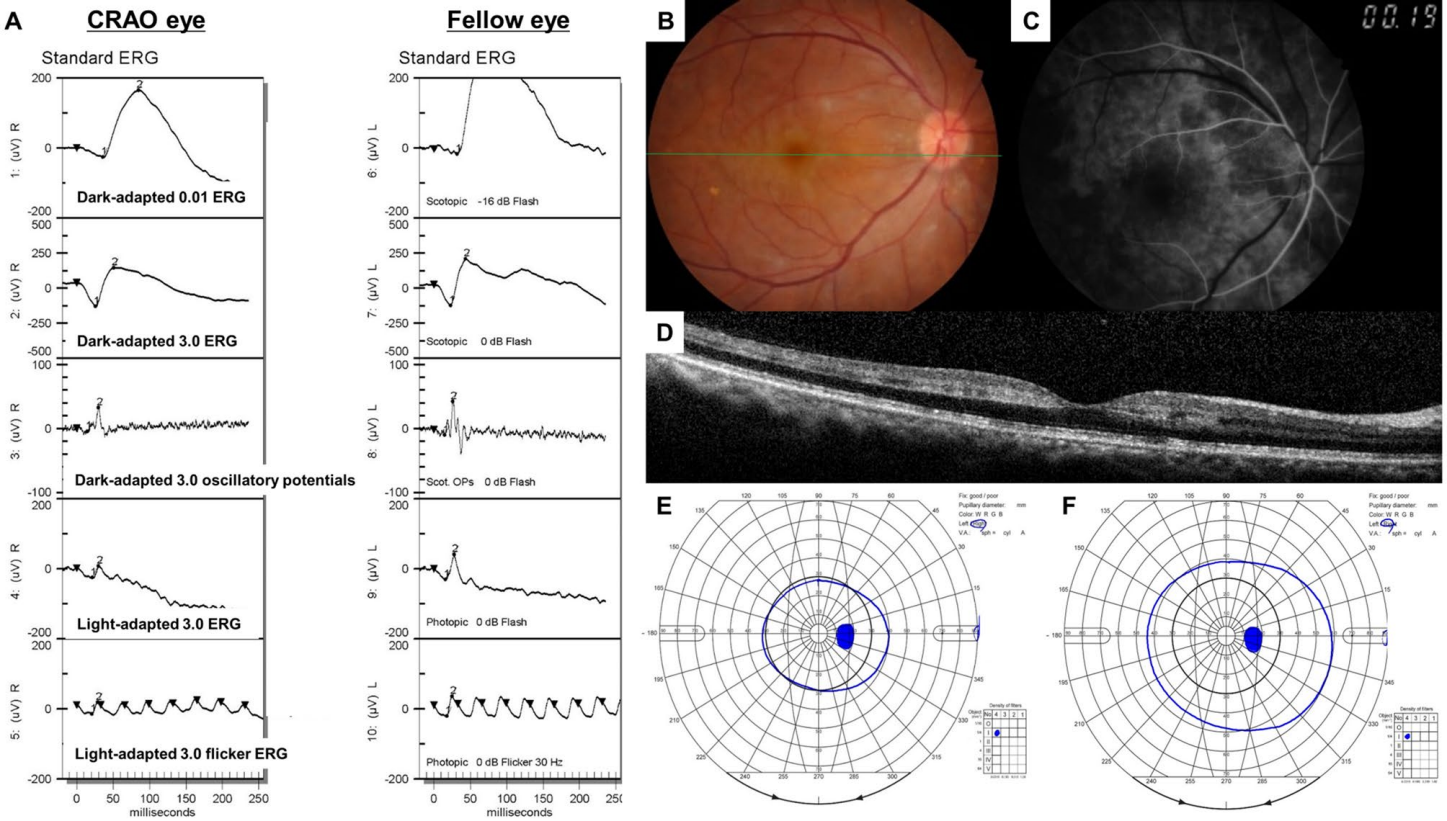
Representative figures of a patient diagnosed with incomplete CRAO in the right eye. Green line in the fundus photo indicates the scanning line of SD-OCT. (**A**) ERG pattern of the CRAO eye (right eye) and the fellow uninvolved eye (left eye). Slight decrease in the B-wave amplitude of the CRAO eye compared to the fellow uninvolved eye in the dark-adapted 3.0 ERG can be observed. Moreover, PhNR amplitude is depressed in the eye with CRAO compared to the fellow unaffected eye. (**B–D**) Fundus photo, FA and OCT images of the CRAO eye. FA arm-to-retina time was 19 s and OCT features inner retinal hyperreflectivity. Mild hypoperfusion was observed. (**E**, **F**) Plotted visual field at the initial and final visit. Notice that there was peripheral constriction only at the baseline and the visual field was enlarged at the final examination, suggesting visual field improvement.

**Figure 3:**
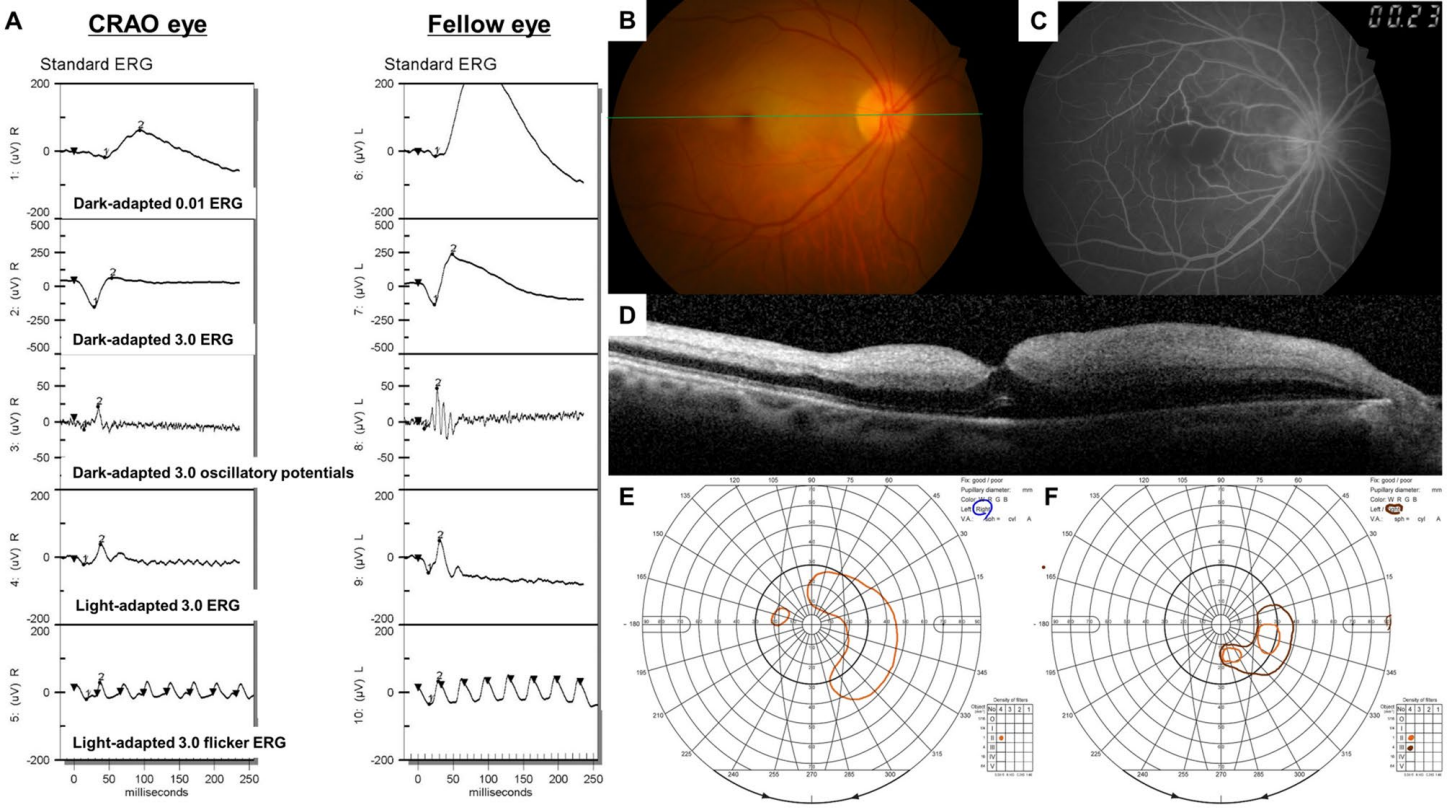
Representative figures of a patient diagnosed with subtotal CRAO in the right eye. Green line in the fundus photo indicates the SD-OCT scanning line. (**A**) ERG pattern of the CRAO eye (right eye) and the fellow uninvolved eye (left eye). B-wave amplitude in both dark-adapted and light-adapted responses and PhNR amplitudes decreased considerably in the subtotal CRAO, compared to the fellow uninvolved eye. (**B**–**D**) Fundus photo, FA and OCT images of the CRAO eye. FA arm-to-retina time was 24 s and OCT features moderate and diffuse inner retinal edema with small subretinal fluid. Severe hypoperfusion was observed. (**E**, **F**) Plotted visual field at the initial and final visit. Notice that there were nasal and temporal islands at the baseline and the visual field was constricted at the final examination, suggesting no visual field improvement.

**Figure 4:**
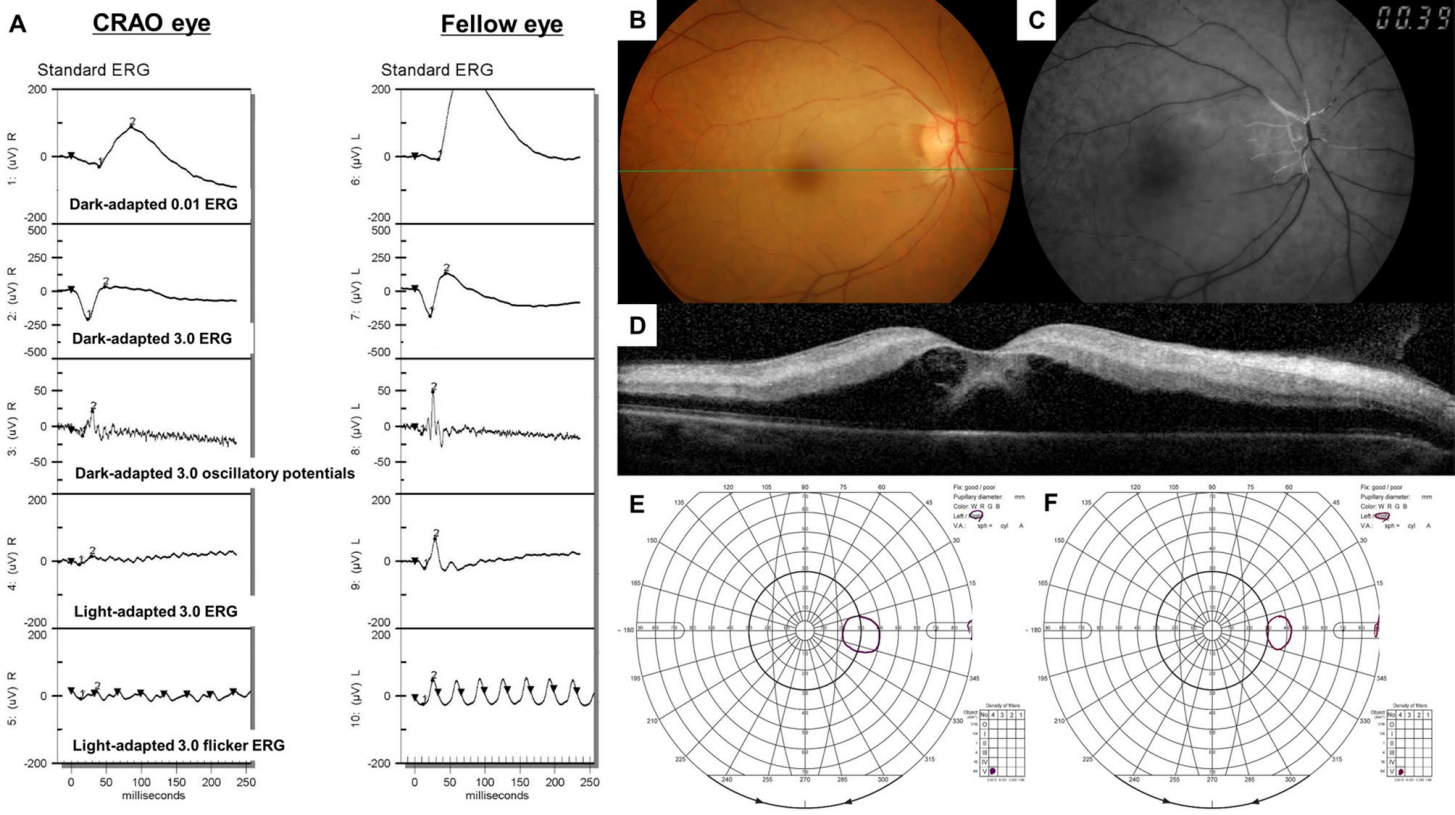
Representative figures of a patient diagnosed with total CRAO in the right eye. Green line in the fundus photo indicates the SD-OCT scanning line. (**A**) ERG pattern of the CRAO eye (right eye) and the fellow uninvolved eye (left eye). Compared to the ERG pattern in Fig. 3, more diminished responses in dark-adapted and light-adapted ERG, including PhNR amplitude, were observed in this case of total CRAO. (**B**–**D**) Fundus photo, FA and OCT images of the CRAO eye. FA arm-to-retina time was 39 s and OCT features severe diffuse inner retinal edema, subretinal fluid, and loss of retinal layers. Retinal perfusion was confined to the optic disc area. (**E**, **F**) Plotted visual field at the initial and final visit. Notice that there was small temporal island at the baseline and the visual field was constricted at the final examination, suggesting no visual field improvement.

For this study, 110 unilateral acute non-arteritic CRAO eyes met the inclusion criteria and were included (Fig. 1). We summarized the specific demographics and clinical characteristics of the analyzed subjects in Table 1. The patients were classified according to the severity of CRAO^6^: incomplete, 30 (27%); subtotal, 57 (52%) and total CRAO, 23 (21%) (Fig. 1). The baseline and final BCVAs, and initial VFDs were statistically different among the CRAO subgroups (all *P* < 0.001, Table 1), while the tendency of declining VA and VFDs were observed according to the stages of CRAO (all *P* < 0.001, Table 1). The following post-hoc analyses suggested that the baseline and final BCVAs were statistically different among CRAO subgroups (all *P* < 0.001). However, for FA arm-to-retina time, statistically significant difference between incomplete and subtotal CRAO was not observed (*P* = 0.503). The representative cases of each CRAO subgroup including ERG, fundus photography, FA, SD-OCT and Goldmann perimetry examinations are described in Fig. 2 (incomplete), Fig. 3 (subtotal), and Fig. 4 (total).

**Table 1:** Demographics and clinical characteristics of CRAO patients and comparisons based on the disease stage.

Variables	All CRAO (N = 110)	Incomplete (N = 30)	Subtotal (N = 57)	Total (N = 23)	P*†‡	Incomplete vs Subtotal	Incomplete vs Total	Subtotal vs Total
Mean age (year)	60.8 ± 15.6 (17, 87)	59.4 ± 14.8 (26, 82)	61.2 ± 15.1 (17, 87)	61.9 ± 18.2 (19, 82)	.806^†^			
Male: Female	67 : 43	22 : 8	33 : 24	12 : 11	.235*			
Standard treatment: Intra-arterial thrombolysis	36 : 74	13 : 17	16 : 41	7 : 16	.341*			
Mean time from symptom onset to treatment (hour)	25.1 ± 35.8 (1, 168)	42.1 ± 51.8 (1, 168)	19.1 ± 26.5 (1, 168)	18.8 ± 24.4 (1, 100)	**.026**‡	**.005**	**.022**	.999
Mean follow-up period (month)	15.7 ± 14.1 (1, 98)	17.4 ± 15.2 (1, 74)	14.1 ± 16.3 (1, 98)	10.2 ± 8.9 (2, 38)	.264^‡^			
FA arm-to-retina time (sec)	23.9 ± 8.9 (11, 55)	21.0 ± 6.6 (11, 42)	22.9 ± 7.5 (11, 47)	29.8 ± 11.5 (13, 55)	** < .001**†	.503	** < .001**	** < .001**
**Visual acuity (logMAR)**								
Mean baseline BCVA	2.17 ± 0.52 (20/90, NLP)	1.64 ± 0.68 (20/90, HM)	2.26 ± 0.22 (20/1,000, NLP)	2.59 ± 0.23 (HM, NLP)	** < .001**‡	** < .001**	** < .001**	** < .001**
Mean final BCVA	1.84 ± 0.78 (20/18, NLP)	0.97 ± 0.77 (20/18, HM)	2.01 ± 0.48 (20/300, HM)	2.52 ± 0.27 (FC, NLP)	** < .001**‡	** < .001**	** < .001**	** < .001**
Visual acuity improvement^¶^	50/110 (46%)	21/30 (70%)	26/57 (46%)	3/23 (13%)	< .001*			
**Baseline visual field defects**^**§**^								
Mild : Severe VFDs	43 : 67	26 : 4	16 : 41	1 : 22	< .001*			
Visual field improvement^#^	25/54 (46%)	15/20 (75%)	10/27 (37%)	0/7 (0%)	< .001*			

*P* values in boldface indicate statistical significance.

*Pearson’s Chi-Square test.

^†^One-way ANOVA for continuous parametric variables.

^‡^Kruskal–Wallis test and Mann–Whitney U test for continuous nonparametric variables.

^¶^Visual acuity improvement was evaluated as the improvement in final BCVA compared to baseline BCVA.

^§^Mild visual field defect includes peripheral constriction, paracentral scotoma, central and cecocentral scotoma, and severe visual field defect includes temporal island and no visual field.

^#^Visual field improvement was evaluated in only those that underwent at least two sequential visual field tests (N = 54).

*CRAO* central retinal artery occlusion, *FA* fluorescein angiography, *logMAR* logarithm of the minimal angle of resolution, *FC* finger count, *HM* hand motion, *NLP* no light perception, *BCVA* best-corrected visual acuity, *VFD* visual field defect.

### Electroretinography (ERG) in CRAO patients

**Figure 5:**
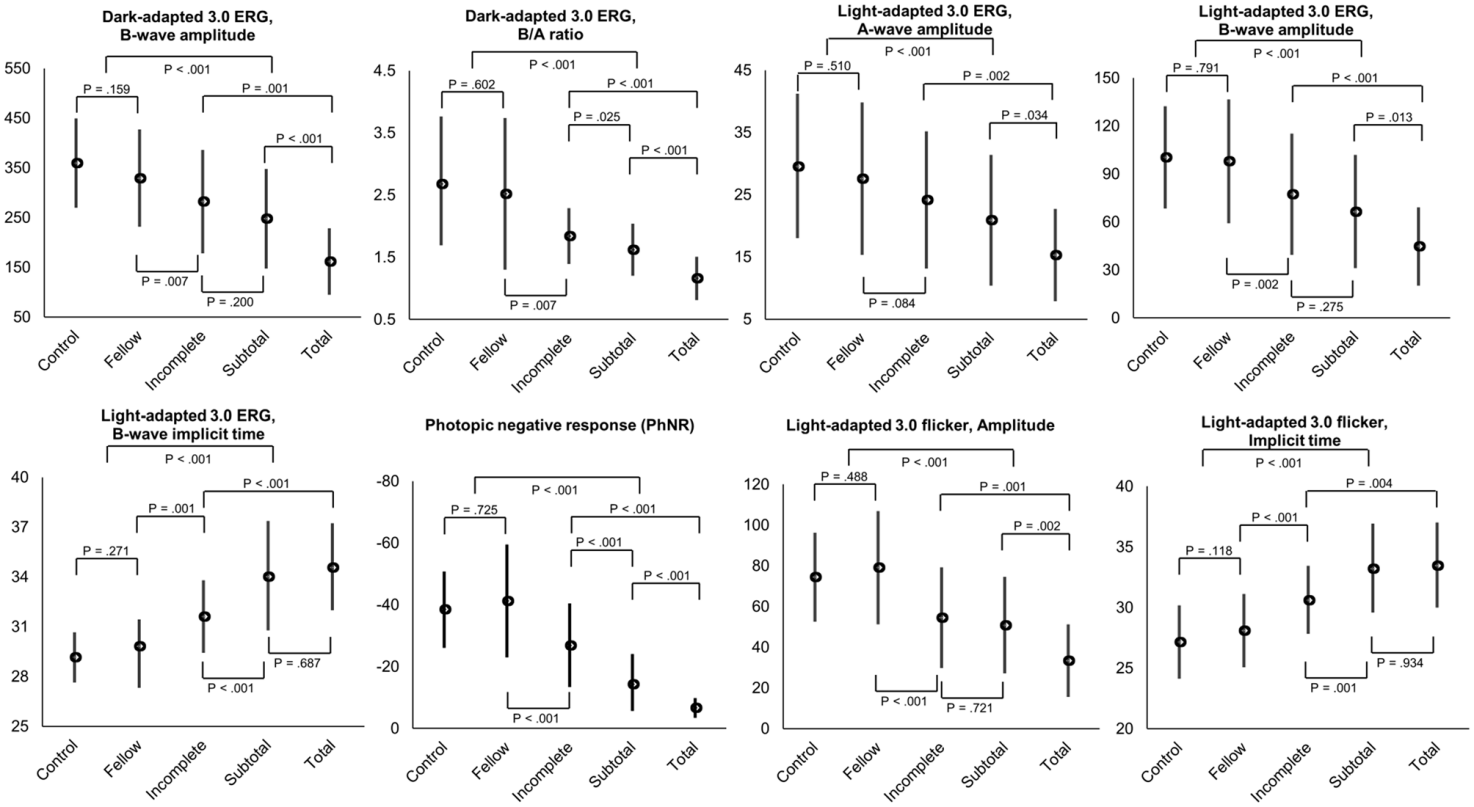
Bar graphs showing the association of ERG parameters and CRAO eyes (based on the disease stage), fellow uninvolved eyes, and control eyes. Detailed data are summarized in Table 2, and in this figure, we plotted bar graphs with relevant ERG parameters to visualize the tendency of ERG changes according to the severity of retinal ischemia.

The associations of ERG parameters and CRAO eyes, fellow uninvolved eyes, and normal controls are summarized in Table 2. Before statistical analysis, we checked whether the treatment options had affected the ERG parameters in each CRAO subgroup and observed no significant differences in ERG parameters according to the treatment choices (standard treatment vs. intra-arterial thrombolysis). Compared to the fellow uninvolved eyes, CRAO eyes were significantly different in all ERG components except dark-adapted 3.0 A-wave amplitude (all *P* < 0.001, except *P* = 0.887). We set the age and sex-matched controls of 30 normal eyes, which were enrolled as the healthy patients with routine ophthalmologic examinations, and the results showed no differences compared to the fellow uninvolved eyes. There were significant differences among CRAO subgroups in dark-adapted 0.01 B-wave amplitude (*P* = 0.002), dark-adapted 3.0 B-wave amplitude and B/A ratio (all *P* < 0.001), light-adapted 3.0 A-wave amplitude (*P* = 0.003), B-wave amplitude and B-wave implicit time (all *P* < 0.001), photopic negative response (PhNR) amplitude (*P* < 0.001), and light-adapted 3.0 flicker response amplitude and implicit time (all *P* < 0.001). The post-hoc analyses were performed on the CRAO subgroups (incomplete, subtotal, and total): B/A ratio and PhNR amplitude were significantly different in each subgroup (*P* = 0.025 and < 0.001, all *P* < 0.001, respectively), while dark-adapted 0.01 B-wave amplitude, dark-adapted 3.0 B-wave amplitude, light-adapted 3.0 A-wave amplitude and B-wave amplitude, light-adapted 3.0 flicker response amplitude were significantly different only between total and other CRAOs. However, there were no differences between the incomplete and subtotal subgroups (detailed data in Table 2). In addition, light-adapted 3.0 B-wave implicit time and light-adapted 3.0 flicker response implicit time were significantly different only between incomplete and other subgroups, but no difference between the subtotal and total subgroups (detailed data in Table 2). The A-wave amplitude and implicit time in dark-adapted 3.0 ERG did not differ among the CRAO subgroups, however the linear trend was suggested in A-wave implicit time in dark-adapted 3.0 (*P* for linear trend = 0.057) and A-wave amplitude in light-adapted 3.0 ERG (*P* for linear trend = 0.001). Overall, the 8 ERG parameters that are significantly different among CRAO subgroups are plotted as bar graphs for visualization (Fig. 5).

**Table 2:** Association of ERG parameters and CRAO eyes (based on the disease stage), Fellow uninvolved eyes, and Normal Controls.

Parameters	All CRAO (N = 110)	Incomplete (N = 30)	Subtotal (N = 57)	Total (N = 23)	CRAO subgroups P*†‡	Incomplete vs Subtotal	Incomplete vs Total	Subtotal vs Total	Fellow eyes (N = 110)	Involved vs Fellow *P*^§^	Control eyes (N = 30)	Control vs Fellow eyes *P*^#^
**Dark-adapted 0.01**												
B-wave amplitude (μV)	135.09 ± 91.98 (5.3, 411.3)	149.17 ± 85.11 (16.9, 344.1)	125.18 ± 99.99 (13.0, 411.3)	69.90 ± 56.98 (5.3, 220.0)	**.002**†	.394	**.001**	**.015**	199.56 ± 117.10 (13.1, 443.5)	** < .001**	234.74 ± 98.65 (61.7, 409.7)	.080
Implicit time (ms)	90.99 ± 16.48 (35.5, 133.1)	91.26 ± 15.69 (66.0, 130.0)	95.40 ± 19.05 (35.5, 138.1)	97.47 ± 19.32 (42.5, 128.7)	.370†	.521	.370	.866	86.16 ± 16.92 (55.5, 135.6)	** < .001**	80.17 ± 11.12 (60.0, 94.0)	.111
**Dark-adapted 3.0**												
A-wave amplitude	159.49 ± 67.72 (26.2, 334.3)	160.87 ± 66.54 (52.3, 334.3)	158.63 ± 63.75 (43.9, 327.3)	143.76 ± 66.37 (26.2, 281.2)	.515†	.985	.549	.558	155.24 ± 72.99 (16.8, 336.7)	.887	150.02 ± 61.05 (81.2, 257.2)	.767
A-wave implicit time	24.66 ± 2.83 (18.0, 32.5)	23.83 ± 2.28 (19.0, 28.0)	24.75 ± 2.80 (18.0, 32.5)	25.45 ± 3.22 (19.3, 32.0)	.066†	.229	.056	.148	22.62 ± 2.18 (16.3, 31.0)	** < .001**	21.38 ± 2.76 (16.3, 26.5)	.094
B-wave amplitude	244.33 ± 106.95 (17.3, 582.3)	282.20 ± 103.93 (124.0, 529.0)	248.26 ± 99.66 (107.0, 582.3)	161.60 ± 66.96 (17.3, 260.3)	** < .001**†	.200	** < .001**	** < .001**	329.10 ± 97.69 (85.7, 531.0)	** < .001**	359.77 ± 89.67 (221.5, 527.5)	.198
B-wave implicit time	50.73 ± 7.05 (34.0, 71.5)	50.45 ± 4.61 (41.3, 63.0)	50.95 ± 7.42 (34.0, 65.5)	50.86 ± 7.04 (38.5, 71.5)	.937†	.932	.969	.998	46.48 ± 4.42 (35.5, 59.0)	** < .001**	44.05 ± 7.05 (29.0, 53.6)	.159
B/A ratio	1.59 ± 0.48 (0.64, 2.85)	1.84 ± 0.45 (1.08, 2.77)	1.62 ± 0.42 (0.83, 2.85)	1.16 ± 0.35 (0.64, 2.23)	** < .001**‡	**.025**	** < .001**	** < .001**	2.52 ± 1.22 (1.20, 7.62)	** < .001**	2.68 ± 0.99 (1.10, 5.27)	.602
**Dark-adapted 3.0**												
Oscillatory potentials	36.25 ± 21.43 (4.1, 92.3)	35.49 ± 21.12 (9.2, 92.3)	34.25 ± 23.14 (4.1, 90.1)	24.32 ± 15.32 (4.3, 71.5)	.085‡	.538	.053	.081	50.53 ± 28.95 (6.1, 144.1)	** < .001**	56.97 ± 28.35 (21.1, 118.1)	.364
**Light-adapted 3.0**												
A-wave amplitude	20.81 ± 10.38 (0.8, 54.9)	24.15 ± 11.01 (6.2, 50.6)	20.90 ± 10.50 (4.2, 54.9)	15.28 ± 7.41 (0.8, 32.0)	**.003**†	.268	**.002**	**.034**	27.58 ± 12.25 (0.1, 67.7)	** < .001**	29.56 ± 11.67 (16.0, 64.0)	.510
A-wave implicit time	16.31 ± 3.13 (6.0, 27.5)	15.65 ± 2.76 (6.0, 21.5)	16.75 ± 3.30 (10.5, 27.5)	16.89 ± 2.52 (12.0, 23.5)	.158†	.187	.233	.976	15.32 ± 1.84 (11.5, 25.5)	** < .001**	15.01 ± 1.54 (13.0, 18.0)	.484
B-wave amplitude	66.45 ± 37.20 (9.6, 176.8)	77.31 ± 37.94 (23.8, 147.5)	66.41 ± 35.40 (6.9, 176.8)	44.74 ± 24.43 (12.0, 93.2)	** < .001**†	.275	** < .001**	**.013**	97.93 ± 38.70 (27.4, 225.2)	** < .001**	100.40 ± 31.97 (50.3, 148.4)	.791
B-wave implicit time	33.66 ± 3.27 (26.0, 47.0)	31.61 ± 2.19 (26.0, 36.0)	34.02 ± 3.35 (28.5, 47.0)	34.56 ± 2.67 (29.0, 40.0)	** < .001**†	** < .001**	** < .001**	.687	29.82 ± 2.51 (14.5, 41.0)	** < .001**	29.16 ± 1.51 (25.5, 31.5)	.271
**Photopic negative response**	− 25.76 ± 11.82 (− 57.2, − 4.8)	− 31.82 ± 13.58 (− 57.2, − 14.2)	− 17.31 ± 9.72 (− 32.9, − 8.4)	− 7.64 ± 3.21 (− 11.7, − 4.8)	** < .001**†	** < .001**	** < .001**	** < .001**	− 47.27 ± 18.25 (− 76.4, − 19.2)	** < .001**	− 40.49 ± 12.41 (− 67.3, − 17.6)	.725
**Light-adapted 3.0 flicker**												
Amplitude	47.98 ± 24.69 (6.7, 113.5)	54.47 ± 24.70 (16.3, 102.3)	50.81 ± 23.71 (7.8, 113.5)	33.41 ± 17.79 (6.7, 64.5)	** < .001**†	.721	**.001**	**.002**	79.07 ± 27.80 (15.9, 156.3)	** < .001**	74.44 ± 21.83 (36.5, 109.7)	.488
Implicit time	32.45 ± 3.59 (24.5, 42.0)	30.61 ± 2.82 (25.1, 35.8)	33.20 ± 3.72 (24.5, 42.0)	33.46 ± 3.48 (26.1, 40.0)	** < .001**†	**.001**	**.004**	.934	28.09 ± 3.02 (23.1, 39.5)	** < .001**	27.16 ± 3.02 (22.1, 37.5)	.118

*P* values in boldface indicate statistical significance.

*P-values compared between each CRAO groups.

^†^One-way ANOVA and student t-test for continuous parametric variables.

^‡^Kruskal–Wallis test and Mann–Whitney U test for continuous nonparametric variables.

^§^P-values compared between CRAO involved eyes and fellow eyes. Paired t-test and Wilcoxon signed rank test for continuous parametric and nonparametric paired variables.

^#^P-values compared to fellow eye. Student t-test for continuous parametric variables and Mann–Whitney U test for continuous nonparametric variables.

*ERG* electroretinogram, *CRAO* central retinal artery occlusion.

**Figure 6:**
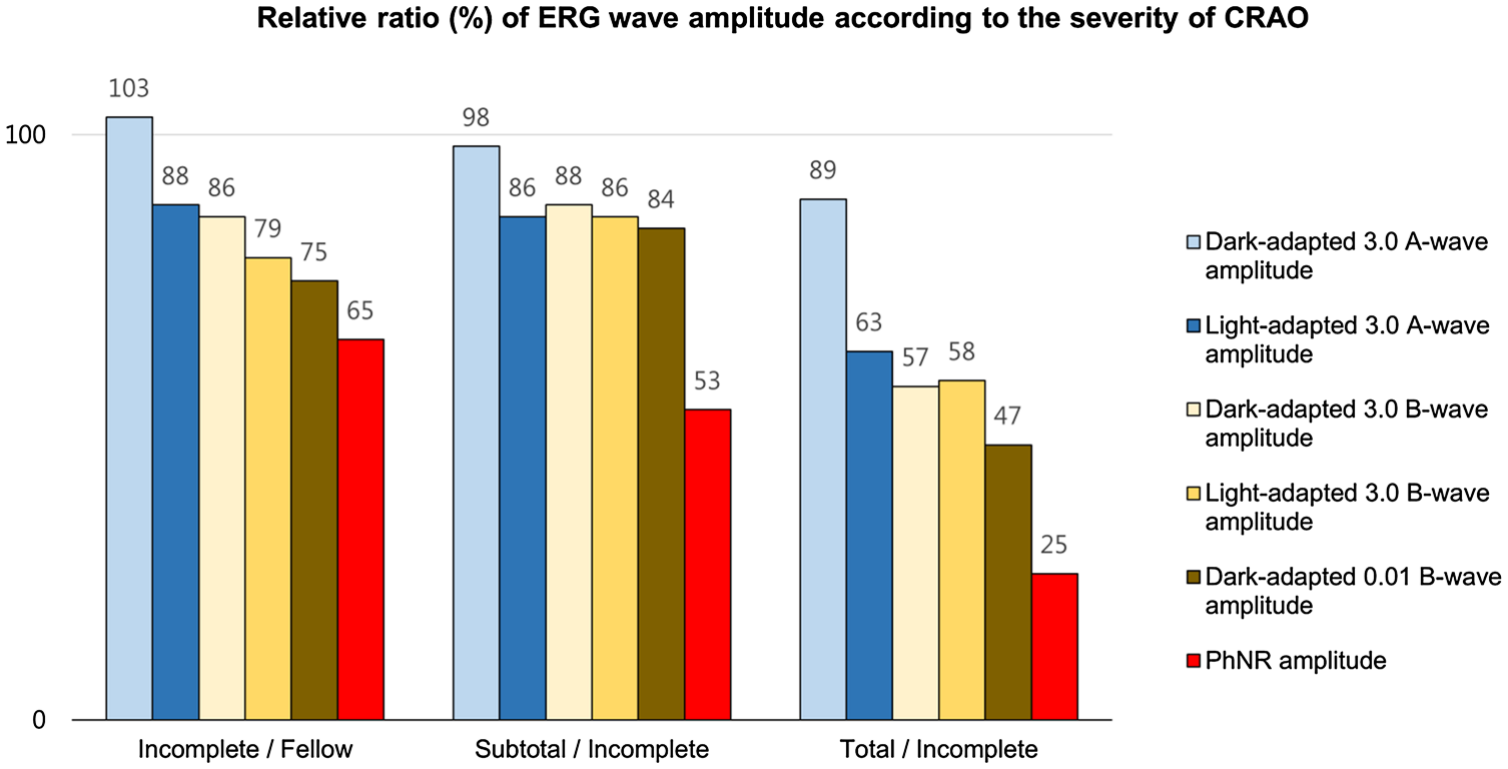
Relative ratio (% percentage) of ERG wave amplitude according to the severity of CRAO. First column describes the percentage ratio of incomplete CRAO compared to the fellow uninvolved eye; to figure out the initially damaged retinal cells and layers. In the early stage of retinal ischemia, PhNR amplitude decreases initially, followed by the dark-adapted 0.01 and light-adapted 3.0 ERG B-wave amplitude. Relatively the A-wave amplitude is preserved. This suggests that the retinal ganglion cells are the most vulnerable in retinal ischemia, and then the interneurons (bipolar cells), while the rods are the least vulnerable. Second and third columns describe the percentage ratio of subtotal and total CRAO compared to the incomplete CRAO. The results show that the PhNR amplitude is the most sensitive indicator for judging the severity of retinal ischemia.

We focused on the ERG wave amplitudes, A-wave and B-wave of dark-adapted 0.01, dark-adapted 3.0, and light-adapted 3.0 ERG, and PhNR, to calculate the relative percentage ratio according to the severity of CRAO (Fig. 6). In eyes with incomplete CRAO, PhNR amplitude showed the largest decrease (65%), while B-wave showed a moderate decrease (75 ~ 86%) and A-wave, the least decrease (dark-adapted 3.0 103%, light-adapted 3.0 88%) compared to the amplitudes of the normal fellow eyes. In subtotal and total CRAO eyes, PhNR amplitude also showed the largest decrease (subtotal 53%, total 25%) and B-wave amplitudes showed a moderate decrease compared to the incomplete CRAO eyes. Although dark-adapted 3.0 A-wave amplitude is quite well preserved in subtotal and total CRAO eyes, the light-adapted 3.0 A-wave showed a significant decrease in total CRAO eyes compared to the incomplete CRAO eyes. In addition, according to Table 2 data, light-adapted 3.0 flicker response amplitude showed a dramatic decrease in total CRAO than in incomplete or subtotal CRAO. Considering that the light-adapted 3.0 A-wave and flicker response represent both the activity of cone photoreceptors and OFF bipolar cells^20^, interneurons and cone photoreceptors are susceptible afterwards the ganglion cells. It is hard to conclude the distinction between the cone response and OFF bipolar cell response from our study alone. However, macular photoreceptor disruption often observed on OCT in very severe CRAO cases (Fig. 4) suggests that the cone photoreceptor function is markedly reduced in severe CRAO eyes. This leads to the conclusion that rod photoreceptors (dark-adapted A-wave) are the least vulnerable retinal cells to spontaneous CRAO.

### Clinical visual function and prognosis in CRAO patients

**Figure 7:**
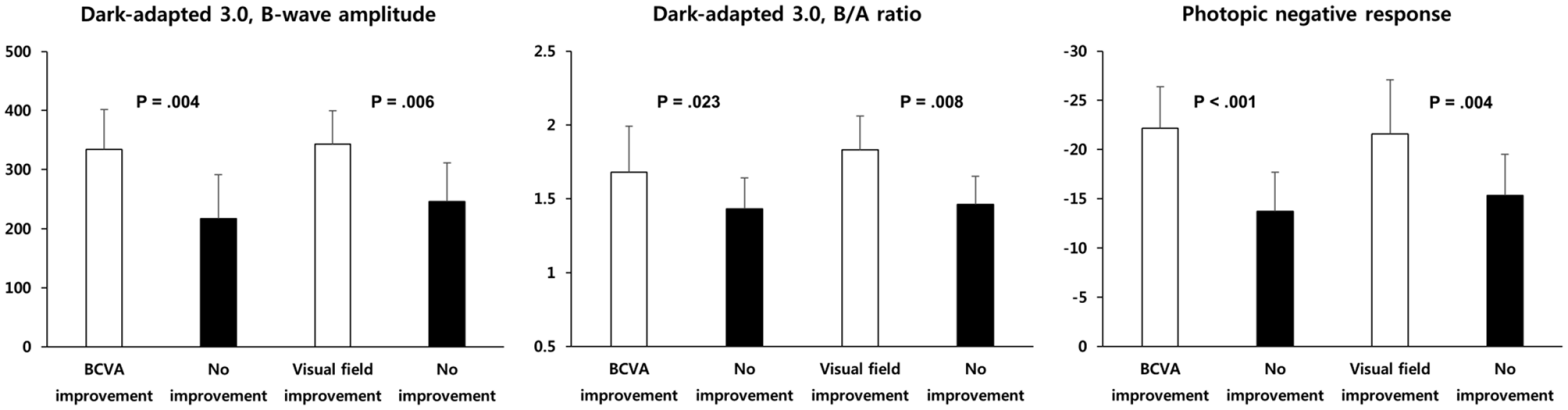
Bar graphs showing the association of ERG parameters and visual prognosis in CRAO eyes. Detailed data is summarized in Table 3, and in this figure, we plotted bar graphs with relevant ERG parameters to visualize the tendency of ERG changes according to the visual acuity and field defect improvement.

The associations of ERG parameters and clinical visual function & prognosis are summarized in Table 3 and Fig. 7. We evaluated clinical visual function by measuring visual acuity and performing visual field tests, and the prognosis was determined by the improvement of visual acuity and visual field defect compared to the baseline data. Briefly, the visual field improvement was considered as both the final quantitative enlargement of the visual field and the patients’ subjective perception. First, B/A ratio showed significant correlations with initial BCVA and VFDs while other ERG parameters did not. Pearson correlation coefficients are − 0.332 (BCVA, *P* < 0.001) and -0.297 (VFDs, *P* < 0.001). Dark-adapted 3.0 B-wave amplitude, B/A ratio, light-adapted 3.0 B-wave implicit time, PhNR amplitude and light-adapted 3.0 flicker response implicit time revealed significant correlation with visual acuity improvement (*P* = 0.004, *P* = 0.023, *P* = 0.007, *P* < 0.001, *P* = 0.010, respectively). Subsequently, dark-adapted 3.0 B-wave amplitude, B/A ratio, light-adapted 3.0 B-wave amplitude and implicit time, PhNR amplitude and light-adapted 3.0 flicker response implicit time showed significant association with baseline VFDs classified as mild and severe VFDs (*P* = 0.002, *P* < 0.001, *P* = 0.025, *P* = 0.006, *P* < 0.001, *P* < 0.001, respectively). Lastly, dark-adapted 3.0 B-wave amplitude, B/A ratio, light-adapted 3.0 B-wave amplitude, PhNR amplitude and light-adapted 3.0 flicker response implicit time appeared to be significantly associated with visual field improvement (*P* = 0.006, *P* = 0.008, *P* = 0.037, *P* = 0.004, *P* = 0.021, respectively). Meanwhile, baseline BCVA was not associated with visual acuity improvement (*P* = 0.089) nor visual field improvement (*P* = 0.056). The main three ERG parameters highly correlated to the visual prognosis are plotted as bar graphs in Fig. 7.

**Table 3:** Association of ERG parameters and clinical visual function & prognosis variables in eyes with CRAO.

Parameters		Initial BCVA	Initial VFDs	Visual acuity improvement (N = 110)		*P*^†‡^	Visual field improvement (N = 54)¶		*P*^†‡^
		P*	*P*^†‡^	BCVA improvement (N = 50)	No improvement (N = 60)		Visual field improvement (N = 25)	No improvement (N = 29)	
Standard treatment : Intra-arterial thrombolysis				17 : 33	19 : 41	.795^#^	7 : 18	7 : 22	.805^#^
Dark-adapted 0.01	B-wave amplitude (μV)	.636	.274^†^	131.45 ± 91.55	109.57 ± 92.13	.174^†^	180.81 ± 96.84	139.92 ± 92.96	.120^†^
	Implicit time (ms)	.597	.661^†^	91.41 ± 17.36	97.52 ± 18.70	.055^†^	87.50 ± 16.47	85.34 ± 15.37	.620^†^
Dark-adapted 3.0	A-wave amplitude	.941	.773^†^	163.51 ± 61.19	149.79 ± 67.58	.227^†^	185.79 ± 73.36	170.84 ± 67.30	.438^†^
	A-wave implicit time	.676	.593^†^	24.41 ± 1.98	24.88 ± 3.35	.310^†^	24.03 ± 2.67	25.07 ± 2.83	.172^†^
	B-wave amplitude	.339	.093^†^	333.98 ± 67.81	217.19 ± 73.85	**.004**^†^	342.92 ± 56.48	245.74 ± 65.30	**.006**^†^
	B-wave implicit time	.843	.779^†^	50.33 ± 5.55	51.19 ± 7.47	.460^†^	50.28 ± 5.58	50.62 ± 7.44	.850^†^
	B/A ratio	** < .001**	** < .001**^‡^	1.68 ± 0.31	1.43 ± 0.21	**.023**^‡^	1.83 ± 0.23	1.46 ± 0.19	**.008**^‡^
Dark-adapted 3.0 Oscillatory potentials		.691	.641^‡^	35.34 ± 21.53	30.01 ± 21.20	.154^‡^	44.43 ± 23.77	40.67 ± 21.45	.544^‡^
Light-adapted 3.0	A-wave amplitude	.250	.289^†^	21.99 ± 11.91	19.33 ± 8.99	.156^†^	10.79 ± 2.15	11.43 ± 2.12	.107^†^
	A-wave implicit time	.812	.952^†^	16.41 ± 2.48	16.56 ± 3.43	.767^†^	15.71 ± 2.20	14.93 ± 2.60	.244^†^
	B-wave amplitude	.344	.087^†^	69.34 ± 34.52	63.28 ± 38.41	.330^†^	83.84 ± 37.68	63.96 ± 36.84	**.037**^†^
	B-wave implicit time	.597	.084^†^	32.71 ± 2.92	34.17 ± 3.19	**.007**^†^	32.42 ± 2.28	33.70 ± 3.16	.090^†^
Photopic negative response		.071	.375^†^	− 22.14 ± 4.25	− 13.72 ± 3.97	** < .001**^†^	− 21.56 ± 5.52	− 15.33 ± 4.18	**.004**^†^
Light-adapted 3.0 flicker	Amplitude	.700	.222	52.55 ± 23.92	44.22 ± 23.57	.052^†^	56.24 ± 23.98	46.79 ± 25.57	.129^†^
	Implicit time	.607	.326	31.69 ± 3.80	33.31 ± 3.32	**.010**^†^	31.07 ± 2.42	33.21 ± 4.03	**.021**^†^

*P* values in boldface indicate statistical significance.

*Correlation analysis.

^†^One-way ANOVA and student t-test for continuous parametric variables.

^‡^Kruskal–Wallis test and Mann–Whitney U test for continuous nonparametric variables.

^¶^Visual field improvement was evaluated in only those underwent at least two sequential visual field tests (N = 54).

^#^Pearson’s Chi-Square Test.

*ERG* electroretinogram, *CRAO* central retinal artery occlusion, *BCVA* best-corrected visual acuity, *VFD* visual field defect.

**Figure 8:**
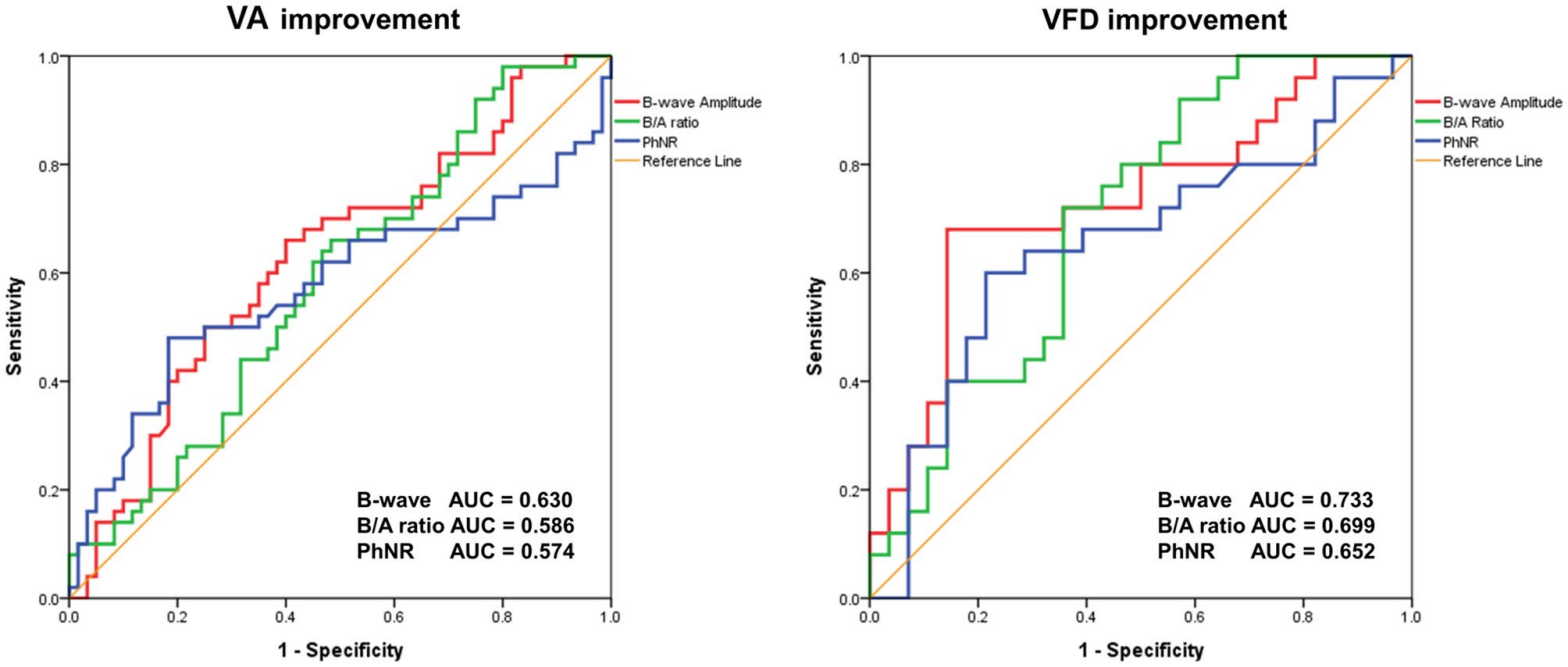
ROC curves presenting the predictive accuracy of ERG parameters, B-wave amplitude in dark-adapted 3.0 ERG (Red), B/A ratio (Green), and PhNR (Blue), in visual acuity and visual field improvement. Combinations of three parameters were also elicited with higher AUCs and significance (not described in this ROC curves figure). In VA improvement, the data is as follows: B-wave amplitude (AUC 0.630, *P* = .020); B/A ratio (AUC 0.586, *P* = .120); PhNR (AUC 0.574, *P* = .181); Combination of three parameters (AUC 0.646, *P* = .009). In VFD improvement, the data is as follows: B-wave amplitude (AUC 0.733, *P* = .004); B/A ratio (AUC 0.699, *P* = .013); PhNR (AUC 0.652, *P* = .058); Combination of three parameters (AUC 0.759, *P* = .001).

In addition, we drew the receiver operating characteristic (ROC) curves derived from the main three ERG parameters, B-wave amplitude in dark-adapted 3.0 ERG, B/A ratio, and PhNR for comparison (Fig. 8). In VA improvement, only B-wave amplitude resulted in statistical significance (*P* = 0.020) with area under the curve (AUC) 0.630, whereas B/A ratio (*P* = 0.120) with AUC 0.586 and PhNR (*P* = 0.181) with AUC 0.574 were withdrawn. The combination of the three parameters showed slightly improved significance (*P* = 0.009) with AUC 0.646. In VFD improvement, B-wave amplitude and B/A ratio resulted in statistical significance (*P* = 0.004) with AUC 0.733 and (*P* = 0.013) with AUC 0.699, whereas PhNR showed *P* = 0.058 with AUC 0.652. The combination of three parameters presented a more improved significance (*P* = 0.001) with AUC 0.759.

## Discussion

The ERG pattern analysis has been accepted for a long time as the evaluation of outer and inner retinal function^12,13^. Over 50 years ago, Karpe’s reports and a study by Henkes first proposed that a highly attenuated B-wave of the ERG and a relatively preserved A-wave are observed in patients with CRAO^10,11^. Henkes referred to this characteristic ERG pattern as electronegative ERG. Since then, later studies have investigated the ERG patterns in CRAO patients, and found that the reduced B-wave and pronounced A-wave might be due to the severe damage of the ON bipolar cells in the inner retinal layer, whereas the photoreceptors, which are perfused by the choroidal circulation via the short posterior ciliary arteries, are rather spared^21–23^. This, in fact, supports the suggestion of duality of the retinal blood supply, in accordance with standard fundus anomalies like inner retinal layer edema and cherry red spot appearance of the macula^24^.

However, it has been suggested that the classical ERG findings do not correspond well to visual acuities and visual fields^16^. Thus, recently, the predominant loss of the photopic negative response (PhNR) in CRAO has been emphasized^16,17^. PhNR is known to reflect the function of retinal ganglion cells (RGCs) and their axons in the inner retina, and nowadays, RGC function is becoming an important indicator because of the vulnerability of the RGC to ischemia^18^. Machida et al.^16^ suggested that the reduction of the PhNR amplitude was more significant in the CRAO affected eyes than that of dark-adapted 3.0 and light-adapted 3.0 ERG B-wave. Moreover, the authors demonstrated that the severely depressed PhNR amplitude is highly correlated to the degree of visual field defects. Matsumoto et al.^17^ pointed out that the PhNR amplitude is more reduced according to the severity of retinal ischemia in CRAO eyes.

Our results showed that the several ERG parameters have significant correlation to the severity of retinal ischemia. Precisely, only two components of ERG, B/A ratio and PhNR amplitude, are revealed as significantly different among the CRAO subgroups: incomplete, subtotal, and total. These results are consistent with the previous studies, which highlight both B/A ratio and PhNR amplitude as the representative indicators of inner retinal layer function^15–17,23^. In addition, we investigated the clinical visual function and prognosis associated with the ERG components recorded. Previously, Yotsukura and Adachi-Usami^15^ described the result of a correlation between an increase in dark-adapted 0.01 B-wave amplitude and visual recovery in 15 CRAO patients. Our study included 110 unilateral CRAO patients who underwent both ERG and Goldmann perimetry; and we selected 54 patients who performed sequential visual field tests after an interval of at least 6 months, for evaluation of visual field prognosis. The results showed that the baseline BCVAs have significant correlation with only B/A ratio. However, in terms of visual prognosis, there were significant differences in several ERG parameters (Table 3). Notably, ERG components of dark-adapted 3.0 B-wave amplitude, B/A ratio, and PhNR amplitude were interpreted as the indicators of both visual acuity and visual field improvement. ROC curves suggest that among the correlated ERG parameters, B-wave amplitude in the dark-adapted 3.0 ERG is the best predictive component in visual prognosis, while the combination of the three main correlated parameters increases the significance and predictive accuracy. In conclusion, this study provides helpful evidence that the three main ERG components—B-wave amplitude, B/A ratio, and PhNR amplitude—would be a good standard not only for judging the severity of retinal ischemia but for also predicting visual function prognosis.

According to our study, in the early stage of CRAO, inner retinal layer ischemia begins, and both RGCs and the interneurons (bipolar cells) indicated with photopic negative response (PhNR) and light-adapted 3.0 B-wave respectively, are affected initially. Both bipolar cells and retinal ganglion cells are more vulnerable to retinal arterial occlusion than the outer retinal components, since the outer layers are well placed anatomically in relation to the choroidal oxygen source. McLeod suggested that in the oxygenation-based hypoperfusion, oxygen tension (pO_2_) gradients are lower in the middle retina comprised of interneurons (bipolar cells) than in the superficial inner retina (RGCs) that are perfused with retinal arteries and the subsidiary macular arteries^25^. It is assumed that interneurons within the oxygen watershed areas are affected initially by the ischemic insult, which then spreads to the retinal ganglion cell complex. However, in our study, the susceptibility is less severe with interneurons than with the RGCs in the electroretinography (Fig. 6), probably because the interneurons are less vulnerable to ischemia due to their lower oxygen consumption or they can survive and restore their function after reperfusion better than ganglion cells. Moreover, prior study by Curcio and Allen^26^ which suggested that the topographic density of RGCs in the central retinal area outnumbers interneurons, supports the higher sensitivity of PhNR wave change in the early stage of CRAO, since more RGCs in the central retina are likely to be involved in the ischemic insult than interneurons. Previously, Hayreh et al. conducted an experimental study on primates, in which the central retinal artery was clamped and unclamped and the residual B-wave and its recovery were tested^27,28^. It is postulated that the duration of clamping time is correlated to the residual B-wave amplitude, and the residual electrical activity indicates oxygenation from the normoxic tissue compartment in the retinal periphery where interneurons are more densely distributed than RGCs^29^. After the unclamping, recanalization and tissue reperfusion begins and electrical revival is seen, which suggests that the less hypo-oxygenated interneurons could be structurally intact following reperfusion.

In our investigation, A-wave amplitude in light-adapted 3.0 and A-wave implicit time in dark-adapted 3.0 ERG showed a linear trend according to the severity of CRAO, and specifically significant decrease in A-wave amplitude in total CRAO. The A-wave impairment begins from light-adapted 3.0 (cones and OFF bipolar cells) and then progresses to dark-adapted 3.0 (combined) and dark-adapted 0.01 ERG (rods) in extreme central retinal artery occlusion. This finding suggests that in very severe cases, not only the inner retina but also the outer retinal layer could be damaged. Moreover, it is relevant to the previous study with optical coherence tomography, that in severe and total CRAO patients, subretinal fluid (SRF), outer retinal layer dysfunction and even macular photoreceptor disruptions were observed (SRF in subtotal CRAO : 14.1%, total CRAO : 36.4%)^8^.

There are some limitations to this study. Firstly, the present study was performed retrospectively, thus selection bias of treatment choices (standard treatment vs IAT) should not be neglected. According to the data, there were no significant differences in ERG parameters among each CRAO subgroup assigned by treatment options, as mentioned earlier in the results. A possible explanation is that the retinal function did not improve rapidly even after recanalization as the ERG was conducted right after the treatment. Further, there is a possibility that the number of subjects in each subgroup was not sufficient to yield a statistically significant result. Secondly, the symptom onset to treatment time ranged from 1 h to 7 days, which might affect initial ERG and VFD patterns. In addition, only 54 patients (49%) underwent follow-up Goldmann perimetry, even though we tried to maximize the number of subjects enrolled. The excluded subjects may have influenced the results of changes of visual field defects. Moreover, each patient showed variable follow-up periods, which could also affect the final visual evaluation. Lastly, we collected the patients since 2008, therefore, this study followed the ISCEV standard of 2008 update and we did not perform the dark-adapted 10 ERG.

In conclusion, B-wave amplitude in dark-adapted 3.0 ERG, B/A ratio and PhNR amplitude could be determined as the standard indicators for assessing the severity or stage of CRAO, clinical visual function, and prognosis. There is an order of defect in ERG components as the severity of CRAO increases—PhNR, B-wave of light-adapted 3.0 and dark-adapted 3.0, A-wave of light-adapted 3.0, and finally, A-wave of dark-adapted 3.0 response. The ERG data also indicates that ganglion cells and interneurons could be the most vulnerable cells to mild and severe ischemic insult from CRAO. The cones and rod photoreceptors are retinal cells that can be subsequently impaired in very severe ischemic damage from CRAO or ophthalmic artery occlusion.

## Methods

### Patient selection and intervention performed

The institutional review board of Seoul National University Bundang Hospital approved this retrospective study (B-1912/582-101), and the informed consent was waivered by the same institutional committee. This study adhered to the tenets of the Declaration of Helsinki.

Our study enrolled 137 subjects diagnosed with unilateral acute non-arteritic CRAO, who had visited Seoul National University Bundang Hospital between January 2008 and December 2017 (over a 10-year-period) for decreased visual acuity or notable visual field defects. The symptom onset to treatment time was limited to the maximum of 7 days, because of the possible confusion of the ERG pattern due to the time delay. Every patient had undergone fluorescein angiography (FA, VX-10; Kowa OptiMed, Tokyo, Japan), spectral domain optical coherence tomography (SD-OCT, Spectralis OCT; Heidelberg Engineering Inc, Heidelberg, Germany), Goldmann perimetry, and electroretinography (ERG) evaluation.

The exclusion criteria were as follows: ocular trauma history (n = 2); combined ocular pathologies such as retinal vein occlusion, diabetic retinopathy, and macular diseases (n = 17); ocular surgery other than cataract surgery (n = 3); high myopia (> 6 diopters, n = 3); and follow-up duration of less than 1 month (n = 2). Finally, we analyzed 110 eyes from 110 patients with unilateral acute non-arteritic CRAO (Fig. 1). In addition, we included 30 age- and sex-matched normal control subjects who were diagnosed as having no retinal diseases in the same hospital.

As described in our prior studies, patients who suffered acute non-arteritic CRAO were treated with either standard treatments or intra-arterial thrombolysis (IAT)^7,8^. The standard treatments included ocular massage and use of pressure reducing agents. IAT was performed together with cerebral angiography to find out accompanying cerebral vessel diseases. The specific IAT methods were mentioned in our previous studies^7,8^: 500,000 units of urokinase (Green Cross, Yongin, South Korea) injection in the proximal portion of the ophthalmic artery. We selected the patients for IAT treatment in whom visual improvement might be promising, and the characteristics of the subjects are as follows: prolonged retinal arterial perfusion, interval time from symptom onset to treatment of less than 24 h for subtotal or total CRAO patients and less than 1 week for incomplete CRAO patients. Underlying systemic medical conditions of uncontrolled hypertension (systolic blood pressure > 200 mmHg), coagulation disorders, current antithrombotic treatment, and recent history of cerebral infarction/intracranial hemorrhage/myocardial infarction were all excluded for IAT treatment.

### Ophthalmic examinations and electroretinography

At the initial visit and the subsequent follow-up visits, all the enrolled patients underwent clinical ophthalmic evaluations including meticulous examinations by the clinicians, fundus photography, FA, SD-OCT, Goldmann perimetry, and ERG. Best corrected visual acuities (BCVA) were checked with auto refraction. FA arm-to-retina time and arteriovenous passage time were measured for the quantification of the perfusion state^30,31^. OCT images and visual field analyses with Goldmann perimetry were evaluated in the same method as in our previous studies^7–9^. For OCT images, central macular thickness was measured and morphological changes such as retinal thickening, loss of retinal layer structures, and retinal fluid were documented. We performed Goldmann perimetry during each visit, and with reference to our previous study on visual fields, we classified the visual field defects (VFDs) into five representative types in the order of severity—peripheral constriction only, paracentral scotoma, central and cecocentral scotoma, temporal island, and no visual field. Mild VFDs include only peripheral constriction and scotoma, while severe VFDs include temporal island and no visual field. In this study, clinical visual function was evaluated by two criteria—best corrected visual acuity (BCVA) and visual field defect (VFD). Visual prognosis was evaluated in terms of visual acuity and visual field improvement. We set an interval of at least 6 months between the initial visit and the final follow-up to recognize the clinical visual function changes adequately.

The full-field ERG was performed using the UTAS-E2000 System (LKC technologies, Gaithersberg, MD, USA). The recordings of dark-adapted 0.01 (scotopic), dark-adapted 3.0 (combined), dark-adapted 3.0 oscillatory potentials, light-adapted 3.0 (photopic), and light-adapted 3.0 flicker responses were measured following the 2008 standard of the International Society for Clinical Electrophysiology of Vision (ISCEV). In this study, we did not perform the dark-adapted 10.0 ERG. The PhNR amplitude was recorded using the white-on-white stimuli and measured from the baseline to the negative trough between the cone B-waves and the i-waves^16^. The patient’s pupils were fully dilated, the contact lens electrode was put on the cornea, and the reference and ground electrodes were attached on the middle of the forehead and the ear lobe, respectively. All procedures were conducted by one examiner to minimize measurement bias.

### Statistical analyses

According to the severity of retinal ischemia (CRAO stages), we compared demographics and clinical data of BCVAs, VFDs, and ERG parameters. BCVAs were analyzed with the converted values of logarithmic minimum angle of resolution (logMAR). As suggested by Lange et al. ^32^, very low vision of counting fingers or worse, were substituted to logMAR values (finger count: 2.0; hand motion: 2.3; light perception: 2.6; no light perception: 2.9). ERG parameter data were expressed as the mean ± standard deviation. Frequency data were compared using Pearson’s Chi-Square test, and the continuous variables were compared using analysis of variance for parametric data and Kruskal–Wallis test and Mann–Whitney U test for nonparametric data. Pearson’s Chi-Square tests were taken to investigate the associations between CRAO stages and the incidence of visual acuity and visual field improvement. The associations of ERG parameters and CRAO eyes based on the disease stage were analyzed using analysis of variance, student t-test for parametric data and Kruskal–Wallis test and Mann–Whitney U test for nonparametric data. In ERG components, B/A ratio and oscillatory potentials were designated as nonparametric data. The data of CRAO eyes were compared to the data of fellow uninvolved eyes (N = 110) and controls (N = 30). The paired t-test and Wilcoxon signed rank test were performed for the comparison between CRAO eyes and fellow uninvolved eyes, and student t-test for continuous parametric variables and Mann–Whitney U test for continuous nonparametric variables were performed for the comparison between control eyes and fellow uninvolved eyes. In addition, the associations of ERG parameters and clinical visual function and prognosis were investigated subsequently. We adjusted the proportion of standard treatment and intra-arterial thrombolysis treatment group to minimize the effect of treatment choices. The statistical analyses performed in this article were adjusted by Bonferroni method. We performed all statistical analyses using SPSS software version 21.0 for Windows (SPSS, Inc, Chicago, Illinois, USA), and a *P* value less than 0.05 indicated a statistically significant difference.

## Data Availability

The datasets generated and/or analyzed during the current study are available from the corresponding author on reasonable request.
